# Supplementary material for: Determining Reproductive Parameters, which Contribute to Variation in Yield of Olive Trees from Different Cultivars, Irrigation Regimes, Age and Location
Source: Plants (Basel). 2022 Sep 16;11(18):2414. doi: 10.3390/plants11182414 (PMC9504372; doi:10.3390/plants11182414)
Supplement: Supplementary file 1 [file plants-11-02414-s001.zip › plants-1869298-supplementary.pdf]

Supplementary Text file

Contents:

Methods used for olive gene identification and protein alignment 1-2.

Table of Olive genes identified 3.

Supplementary Figures 4-8.

Genomic and putative transcript sequences of the AP2_2 olive group 9-15.

Putative transcript sequences of the AP2_1 olive group.16-18.

Protein sequences of 6 Arabidopsis and 13 putative olive proteins.19-21.

References 21.

Methods used for olive gene identification and protein alignment

The olive OE6 transcript database was searched for sequences encoding proteins similar to Arabidopsis APETALA2 (AP2; AT4G36920) using TBLASTN in the https://denovo.cnag.cat/genomes/olive/blast/ web site. 28 genes with the strongest homology were selected (Supplementary Table 1) and the putative protein sequence of the genes was blasted against the Arabidopsis Araport11 protein dataset, using NCBI BLAST 2.9.0+ BLASTP at TAIR website https://www.arabidopsis.org/Blast/index.jsp. The most similar protein in Arabidopsis was noted ((Supplementary Table 1). The 13 genes encoding similar proteins to either AP2 or TARGET OF EARLY ACTIVATION TAGGED (EAT, TOE1, AT2G28550) were used for further protein alignment using Clustal Omega (Sievers *et al*., 2011) on the EMBL-EBI site https://www.ebi.ac.uk/Tools/msa/clustalo/ the 13 proteins from olive together with 6 Arabidopsis proteins encoding FT transcriptional repressors of the AP2 family were compared. A cladogram of a Neighbour-joining tree without distance corrections was generated (Supplemental Figure 1), noticing two subgroups of olive AP2-like genes most similar to AP2 and TOE1. Primers were designed for each subgroup (Table 2) and specific primers were designed for 3 genes within subgroup AP2_2. (Table 2). Alignment of the protein sequences is presented in Supplementary figure 2. Genomic sequences, expected transcripts and proteins including exon intron borders, primers and MiR172 recognition sites are described for subgroup AP2_2. below.

In genomic sequences, position in the genome is provided, Exons are in CAPS and Bold. The putative ATG translation start site and translation stop codon are underlined and with yellow marking.

In putative transcripts, the putative ATG translation start site and translation stop codon are underlined and with yellow marking. Exon-Exon junctions are with green marking. Consensus Forward (Red) and Reverse (Blue) primer sequences are marked. Specific Forward (double underline) and Reverse (zigzag underline) are marked as well. The miR172 recognition site is in red letters and underlined.

	Olive Transcript	Most similar Arabidopsis Protein	Score (Bits)	E-Value
1	OE6A061030T1	AT4G36920.2 | Symbols: AP2, FL1, AtAP2, FLO2 | FLOWER 1, FLORAL M...	380	7E-128
2	OE6A068128T1	AT4G36920.2 | Symbols: AP2, FL1, AtAP2, FLO2 | FLOWER 1, FLORAL M...	364	3E-123
3	OE6A099997T2	AT4G36920.2 | Symbols: AP2, FL1, AtAP2, FLO2 | FLOWER 1, FLORAL M...	358	2E-120
4	OE6A079258T3	AT4G36920.2 | Symbols: AP2, FL1, AtAP2, FLO2 | FLOWER 1, FLORAL M...	209	8E-65
5	OE6A037406T2	AT2G28550.3 | Symbols: TOE1, RAP2.7 | TARGET OF EARLY ACTIVATION ...	310	7E-101
6	OE6A055418T1	AT2G28550.3 | Symbols: TOE1, RAP2.7 | TARGET OF EARLY ACTIVATION ...	320	5E-105
7	OE6A105872T1	AT2G28550.3 | Symbols: TOE1, RAP2.7 | TARGET OF EARLY ACTIVATION ...	317	2E-103
8	OE6A031451T2	AT2G28550.3 | Symbols: TOE1, RAP2.7 | TARGET OF EARLY ACTIVATION ...	311	1E-102
9	OE6A031619T3	AT2G28550.1 | Symbols: TOE1, RAP2.7 | TARGET OF EARLY ACTIVATION ...	256	2E-80
10	OE6A064439T1	AT2G28550.1 | Symbols: TOE1, RAP2.7 | TARGET OF EARLY ACTIVATION ...	286	2E-92
11	OE6A012609T1	AT2G28550.1 | Symbols: TOE1, RAP2.7 | TARGET OF EARLY ACTIVATION ...	265	1E-83
12	OE6A073355T2	AT2G28550.1 | Symbols: TOE1, RAP2.7 | TARGET OF EARLY ACTIVATION ...	236	1E-72
13	OE6A121142T2	AT2G28550.1 | Symbols: TOE1, RAP2.7 | TARGET OF EARLY ACTIVATION ...	171	8E-49
14	OE6A094680T1	AT1G16060.1 | Symbols: ADAP, WRI3 | ARIA-interacting double AP2 d...	295	4E-98
15	OE6A070880T2	AT1G16060.1 | Symbols: ADAP, WRI3 | ARIA-interacting double AP2 d...	293	1E-97
16	OE6A051383T1	AT4G37750.1 | Symbols: ANT, CKC1, DRG, CKC, AtANT | COMPLEMENTING...	387	2E-127
17	OE6A086517T3	AT4G37750.1 | Symbols: ANT, CKC1, DRG, CKC, AtANT | COMPLEMENTING...	426	3E-144
18	OE6A033132T1	AT4G37750.1 | Symbols: ANT, CKC1, DRG, CKC, AtANT | COMPLEMENTING...	387	2E-127
19	OE6A117716T1	AT4G37750.1 | Symbols: ANT, CKC1, DRG, CKC, AtANT | COMPLEMENTING...	395	4E-132
20	OE6A083451T1	AT1G51190.1 | Symbols: PLT2 | PLETHORA 2 | chr1:18977517-18980305...	568	0E+00
21	OE6A008776T1	AT1G51190.1 | Symbols: PLT2 | PLETHORA 2 | chr1:18977517-18980305...	586	0E+00
22	OE6A108753T1	AT1G51190.1 | Symbols: PLT2 | PLETHORA 2 | chr1:18977517-18980305...	645	0E+00
23	OE6A067815T1	AT5G57390.1 | Symbols: CHO1, PLT5, EMK, AIL5 | PLETHORA 5, EMBRYO...	406	3E-137
24	OE6A040913T1	AT1G72570.1 | Symbols: AIL1 | AP2-like ethylene-responsive transc...	375	2E-125

25	OE6A109454T1	AT1G72570.1 | Symbols: AIL1 | AP2-like ethylene-responsive transc...	371	5E-124
26	OE6A095359T1	AT1G16060.1 | Symbols: ADAP, WRI3 | ARIA-interacting double AP2 d...	243	1E-79
27	OE6A026019T1	AT5G10510.6 | Symbols: AIL6, PLT3 | PLETHORA 3, AINTEGUMENTA-like...	469	4E-161
28	OE6A003667T1	AT5G17430.1 | Symbols: PLT4, BBM | PLETHORA4, BABY BOOM | chr5:57...	398	2E-131

Table S1: A list of Olive genes encoding proteins similar to Arabidopsis proteins with an APETALA2 domain. The olive OE6 transcript database was searched for sequences encoding proteins similar to Arabidopsis APETALA2 (AP2; AT4G36920) using TBLASTN in the https://denovo.cnag.cat/genomes/olive/blast/ web site. 28 genes with the strongest homology were listed and the putative protein sequence of the genes was blasted against the Arabidopsis Araport11 protein dataset, using NCBI BLAST 2.9.0+ BLASTP at TAIR website https://www.arabidopsis.org/Blast/index.jsp. The most similar protein in Arabidopsis was noted.

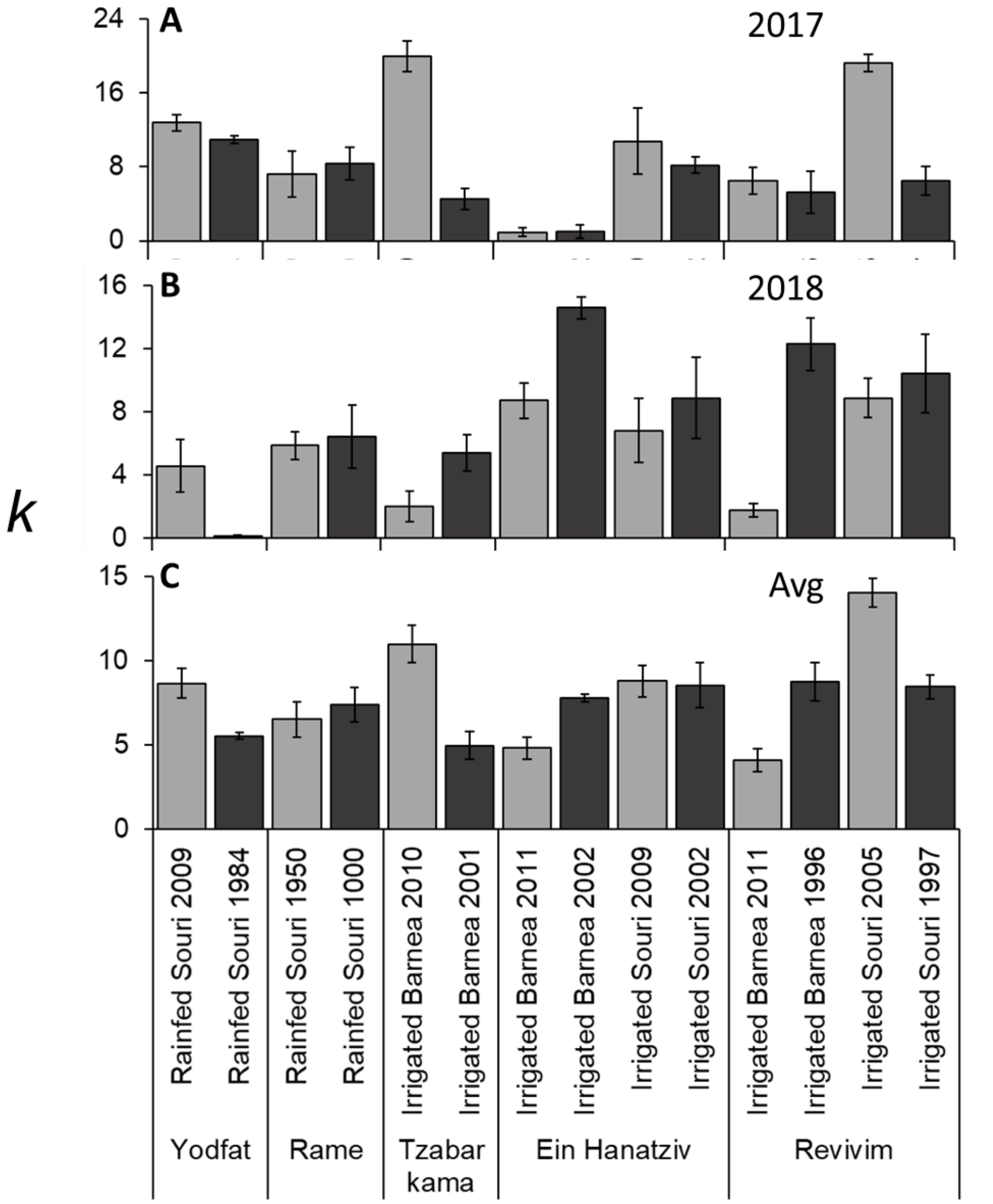

Figure S1. Number of inflorescences per branch (*k*) in different olive groves. First year (**A**), second year (**B**), and average (**C**) values presented. Values are averages of six trees per grove. See materials and methods for measurements. The error bars represent the standard error of the mean for each grove.

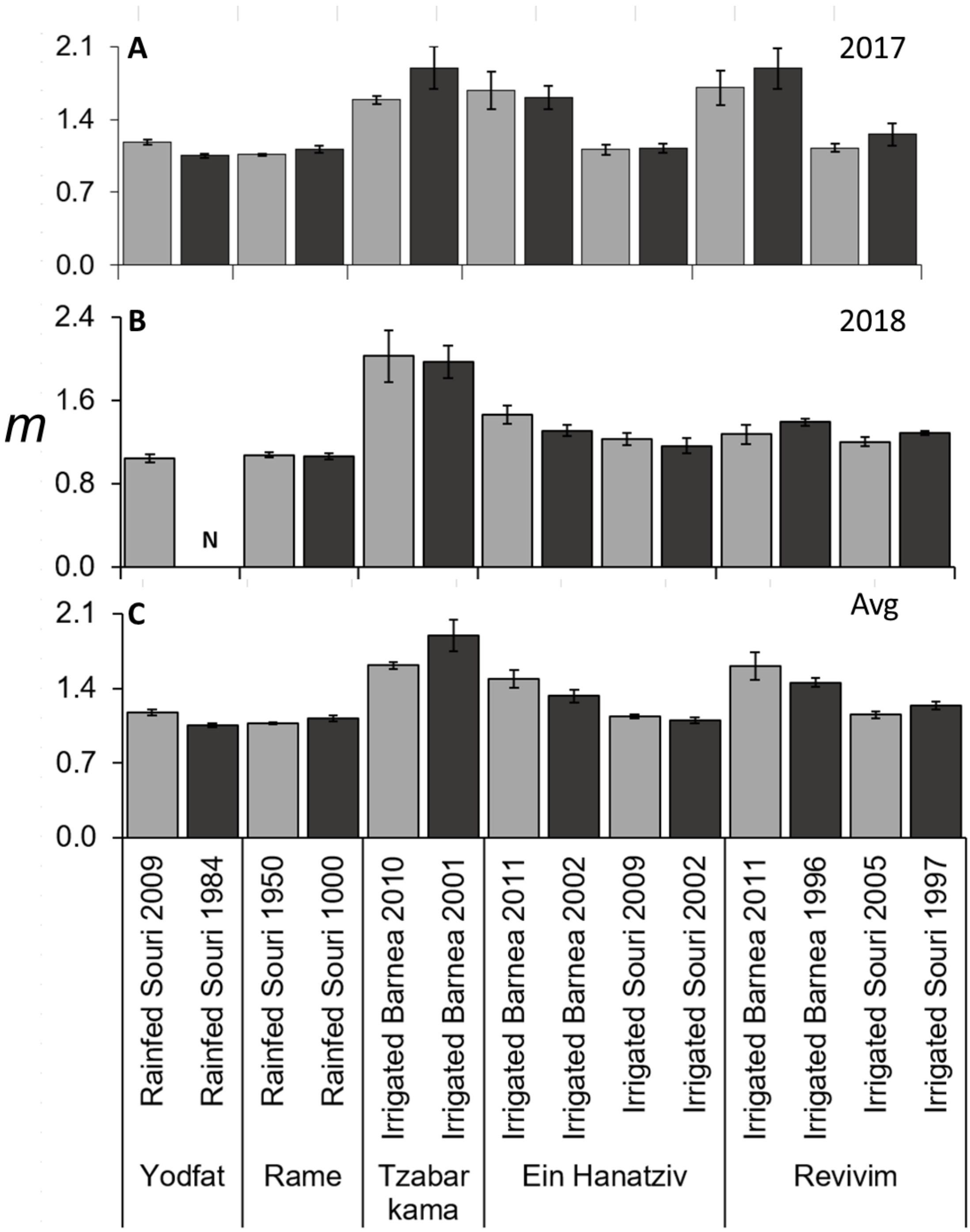

Figure S2. Number of fruits retained on each fruit-bearing inflorescence (*m*) in different olive groves. First year (**A**), second year (**B**), and average (**C**) *m* values presented. Values are averages of six trees per grove. The error bars represent the standard error of the mean for each grove. No significant differences between age groups in the same site (location, cultivar and irrigation method) were identified using the Student's t-test ($P \leq 0.05$).

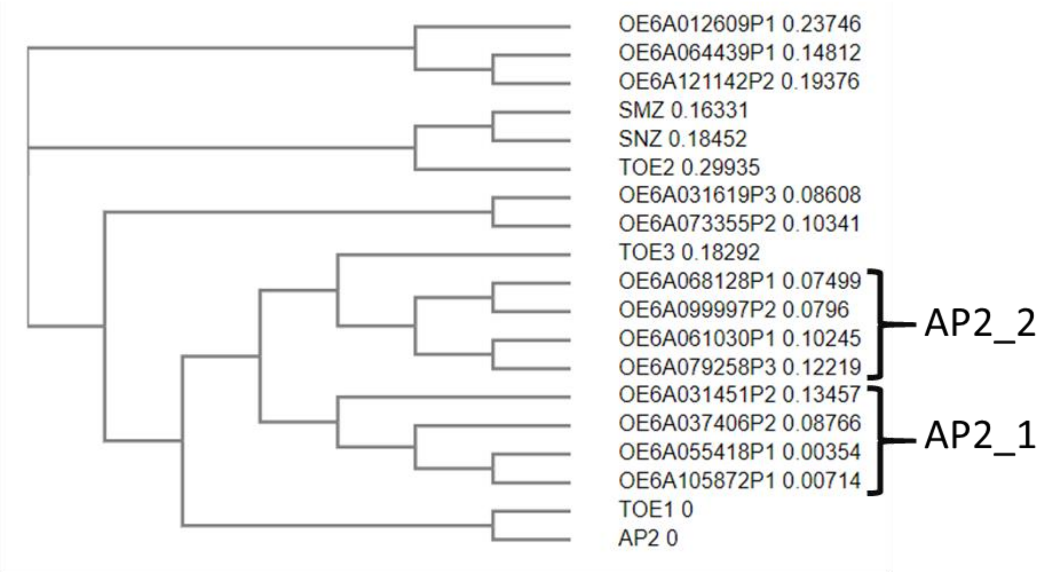

Figure S3. A cladogram of a Neighbour-joining tree without distance corrections for AP2-encoding proteins from Arabidopsis and Olive. 13 putative proteins from olive together with 6 Arabidopsis proteins encoding FT transcriptional repressors of the AP2 family were compared. Nomenclatures for Arabidopsis proteins are AT3G54990 for SMZ, AT2G39250 for SNZ, AT2G28550 for TOE1, AT5G60120 for TOE2, AT5G67180 for TOE3 and AT4G36920 for AP2. Two subgroups (AP2_1 and AP2_2) of olive AP2-like genes most similar to AP2 and TOE1 and analyzed for gene expression are noted. Protein sequences of all proteins compared are in the supplementary text file.

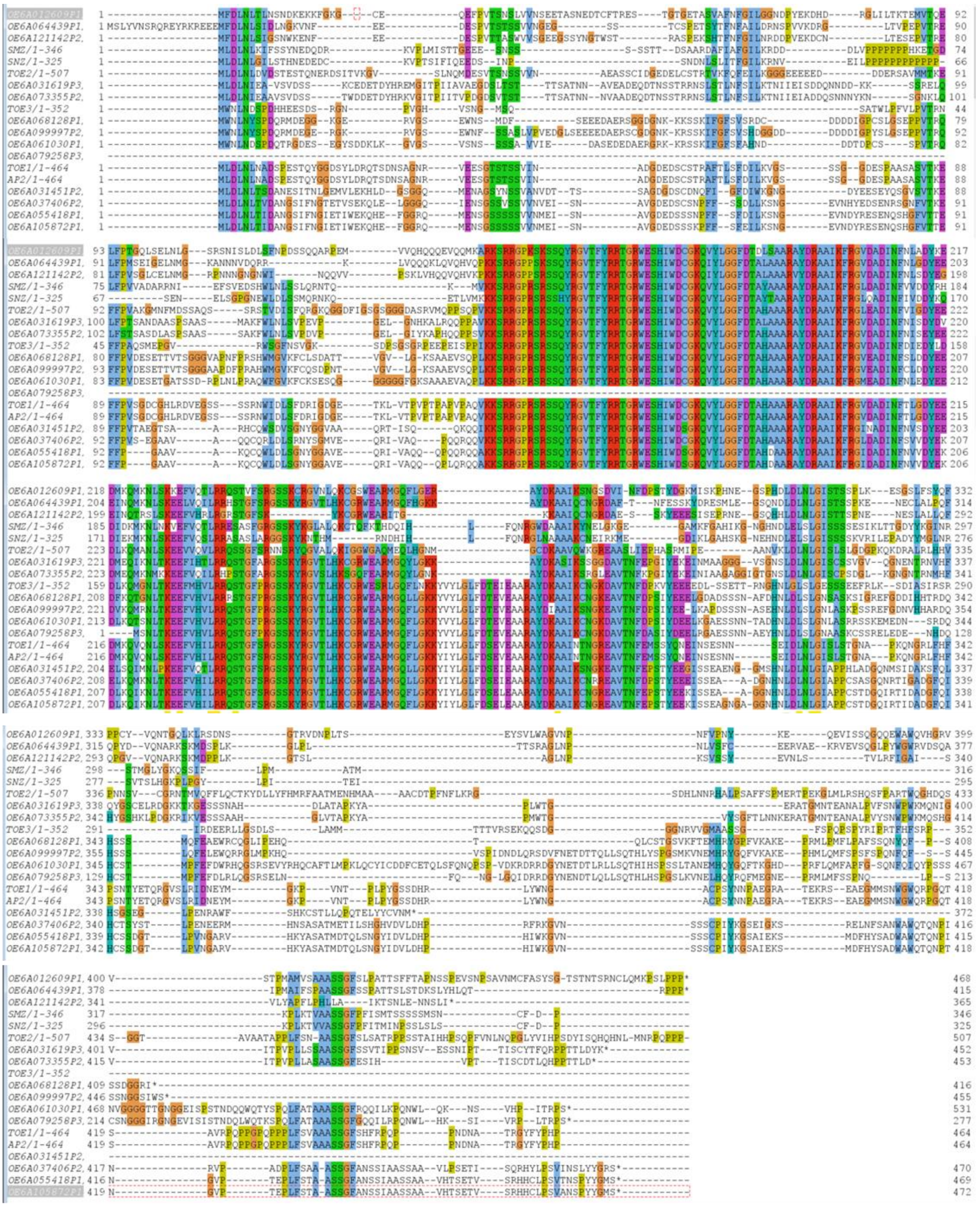

Figure S4. An Amino acid alignment of putative AP2-encoding proteins from Arabidopsis and Olive. 13 putative proteins from olive together with 6 Arabidopsis proteins encoding FT transcriptional repressors of the AP2 family were compared. Nomenclatures for Arabidopsis proteins are AT3G54990 for SMZ, AT2G39250 for SNZ. AT2G28550 for TOE1. AT5G60120 for TOE2. AT5G67180 for TOE3 and AT4G36920 for AP2. Protein sequences of all proteins compared are in the supplementary text file. Alignment was performed using Clustal Omega (Sievers et al., 2011) on the EMBL-EBI site https://www.ebi.ac.uk/Tools/msa/clustalol. Alignment results were viewed in Jalview version 2.11.2.3 with colors assigned using Clustalx.

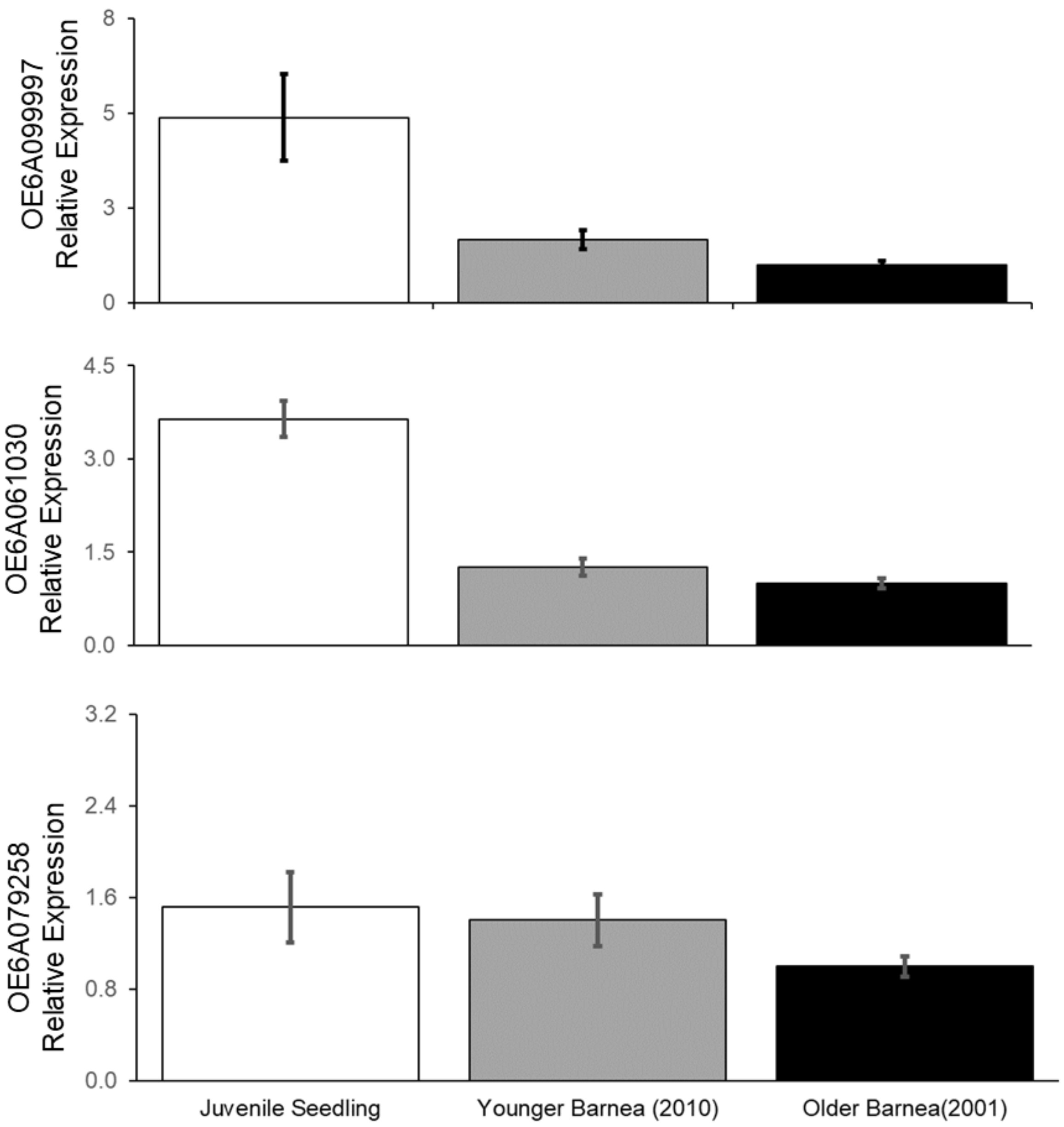

Figure S5. Gene expression of AP2 encoding genes in Leaves of Juvenile and Mature leaves. **A-C** Relative expression levels of genes encoding a subgroup of APETALA2 encoding proteins. See Table 2 and Supplementary text for details of primers and genes. Samples were collected on January 17th 2017 and are identical to those presented in Figure 9 . Relative expression was calculated using qPCR relative to the *OeACT7* gene. Data are based on the means of 5-6 trees per treatment, with the error bars representing the standard error of the mean for each tree type. Juvenile seedlings were of mixed genotype, with 'Barnea' as common female. Mature 'Barnea' trees were planted in different years, as noted.

Group AP2_2 genes

>OE6A099997

Genomic Oe6_s07897:279771..283770 (4 Kb)

gacaagagacgtgagagtgaatgaatgaaaacgaaatgataattaacttagcctaattaaggttattgataaata
agtcaaggttaaggtgttggtgctgtctatttccattattagtgtagttgaagaggtgaaaatgaggagagagaa
agggtattgacatggattgatgcttttcga**TTTGGTTAACGTCGGAAAGTTGAAGGAGGAAGCAAATTGTTATCT**
CTCTCTCTCCCCCCCTCCCTTGCCATTGTTTATCTTTATACGGCCTGTGGAAATAAACAGAGAAAACTTTTAGAG
AGAGAAACTAGAGAGATATAGAGTTAATGTTTCACCTATACATACAGACAGTATTTATGTTACACACAACAAAAT
CCCCTTGAAACTCCAAATCAGCAAGGCAACAAGAGAGATCTAGTTGAATAGGTTTTTTTCTTCTGTCCTTGGGA
AACATAAAATTTTTTCTTTTTCTTTTTACTGCTAGCTACTTGTACAAAACTCAGGCCTTTTGCAGCCCGAAGATT
TTTGCTTCTGATGAGACAGTGCTGATTAGAATTCAGAAAATTTACACAAAACTCTTCACTTTCTCTTTCTAAATT
GTACACTTATCTACAGGGCCTGGCTGAACTGTTCAAATTTCGTTCAAAGAAACAGAAATGTGGAATCTTAACTAT
TCACCGGATCAAAGGATGGATGAAGGTGAAAGGGGTAAACGGGTCGGATCTGAGTGGAACTTTAGCTCAGCATCG
TTGGTGCCGGTGGAGGATGGTTTATCTGAGGAAGAAGAGGACGCAGAGAGAAGTTGTGGTGACGGGAACAAGAAA
AGAAGCAGCAAAATCTTTGGTTTCTCTGTGTCGCATGACGGTGGTGATGATGATGATGATGATATAGGTCCTTAT
TCGTTGGGGAGTGAGCCGCCGGTGACAAGGCAGTTTTTCCCGGTGGACGAGTCGGAAACCACCGTTACATCGGGA
GGTGGAGCAGCACCCGATTTTCCAAGGGCCCACTGGATGGGGGTGAAATTCTGCCAGTCCGACCCCAATACCGTT
GGTGTCTTGGGGAAGTCTGCGGCGGAAGTTTCGCAGCCTCTCAAGAAGAGCCGCCGTGGCCCGAGGTCCCGGAGC
TCTCAGTACCGCGGCGTCACCTTTTACCGGCGAACTGGCCGGTGGGAATCACACATATGgtaacacaatatctaa
atcaacctcaatccttcgtataatcttaccattttcaaaaaaaaatatatttttctgatttttttgtttctaat
tcag**GGATTGTGGAAAACAAGTTTATTTAG**gttagttcatttatcatctctatttataacaagattattcttctt
cttatttaacctgagaattaatgaaactaatggtcttaaataatcagattacccaaattaattattaattttttt
ccctatcttgcaaatttgtatag**GTGGATTTGATACAGCACATGCTGCAGCCCG**gtaagattaactcggattttt
tgcagattcttgattgaattaaaaaataaaaaataaaaattaaatgtgaagttggaagagactaatgcttacaaa
tgaatgtgtgacag**TGCATACGATAGGGCAGCTATTAAGTTTCGTGGAGTAGAAGCAGACATAAACTTCTGTTTG**
GATGATTACGAGGAAGACGTAAAACAGgtagattaatccaagagagacttaggactcaatatgacgaataagatt
ttagcttattttcatctttgctacaaatatgccattaaagtcttctttgttgcaatgtttttgaagagtttgt
attcttgatttagtgttataaattttgaggtttttggacacaggaaaggaaaaatatagtattttccgaggga
tacatgatattctttgtatttcag**ATGAGAAATCTAACGAAGGAAGAATTTGTTCATGTACTTCGACGACAAAGT**
ACTGGTTTCCCAAGAGGAAGTTCCAAGTATAGAGGTGTTACTTTGCACAAATGTGGTAGATGGGAAGCCAGAATG
GGCCAGTTCTTAGGCAAAAAgtaatcacattgtgtccatctctgtgtctgtgtgtgttggtccccaatttctttt
aaatttaaaccatttctgggtttttttcaagtcttaattttgatatataacatcagttgctcttatgtgttaa
cag**GTACGTTTATTTGGGTCTATTTGATACTGAAGTTGAAGCTGCCAG**gtttgtgtggtttagatattatctcca
ttaatttctgcttatatattacctccaactgacagtactggaagcgactgcattgggaatgagattttaacttt
tcccctctaaaatctccgttgagtccggcatatgaaacgtgccaaaagggttaaatctaacttaatttgatggag
ttaattgtcttctgtcaataaattag**GGCTTATGATATAGCTGCCATCAAGAGTAATGGAAAGGAAGCGGTAACC**
AATTTTGATCCCAGCATTTATGAAGAACTTAAAGCTCCAGgtagtttactgttgtgtgtgtgtactttttaaaa
tcttgaattgattctagagatggaaatttttgtgcatgacag**ATTCTTCTAGTAATGCATCTGAGCACAATCTT**
GACTTGAGTTTGGGAAATTTGGCATCAAAACCAAGCAGTCGAGAATTTGGGGATAACGTTCATCATGCTAGAGAC
CAACATTCTTCATCACTGCAATTTGAACTTGAATGGCAACGTCGGGGATTGATGCCGAAGgtatataacgaaggc
atcaatgctttgttaattcaatttgattgaatatcttgaacttgcagtttcttgattttcttgttttgattatcc
gtttcttggtttccag**CACCAAGTGAGTCCGATTGACAATGATTTGCAGAGAAGCGATGTATTCAATGAAACAGA**
TACCACGCAGCTCCTAAGCCAAACGCACCTGTATTCTCCGGGCTCAATGAAGGTTAATGAAATGCATAGATATGG
GCAATTTGTGAAAGCTAAGGAACCCCATATGCTTCAGATGTTTTCACCATCGTTCAGCCCACAAAATTTTCAAgt
aagcccttaatccccgatcctggtattagtaagaattttggcattttcaacgttaatgtgaaaaactgcaatcc
ag**TTTTCAAGCAGCAGCAATGGAGGCAGTATTTGGAGCTGATGGAGTAGAGCTTTCTCTTTATATGAAAGATCAT**
TTATCGCAAACAAATTCTCCTTGATTATTAGCAGCATCATCAGGATTCCCGTGGCTGATAATCGGCTGCAGAAAT
TAAACCGGCCTTCTTTCTGCTATATGCCACTCTAAGATCTCCATTTTTTGGAGTGAGTTTTGTAAAGTTCCATGC
TTTGAGCCTTTTTTGTGCTCAGTGTAACTAGCAGAAACCATTGGTACATGACCAGGCAGGGTTTTCAATTAGGAT
TGAGCACTGAGCAAAAAAACTGGAGAAATTAATATAAAATCCCCAACTTTTCTGGAAATTAATGTTGCTCTTTTG
ATTAATATGTTGTTATAAGTTTAGAGTATATAGGGGATGGAGAATGATTAATGTAACCAACTTCAAATTTATCCA
ACATatttttcttcattcctcattgttttgtttaaacaacgttcataattaatgtctactcttgcattgcatttg
caccagaaaattgaaaaaggaaaaaataaatcctttgaatcagtactcatgagctaaagcagacagacatggtta

ttatgatgagagggacatgacatatgctctaaagcctcgaaggacactgccaaagtgaaaatctctagtattatt
caag

>OE6A099997T2

Putative cDNA

ATGTGGAATCTTAACTATTCACCGGATCAAAGGATGGATGAAGGTGAAAGGGGTAAACGGGTCGGATCTGAGTGG
AACTTTAGCTCAGCATCGTTGGTGCCGGTGGAGGATGGTTTATCTGAGGAAGAAGAGGACGCAGAGAGAAGTTGT
GGTGACGGGAACAAGAAAAGAAGCAGCAAAATCTTTGGTTTCTCTGTGTCGCATGACGGTGGTGATGATGATGAT
GATGATATAGGTCCTTATTCGTTGGGGAGTGAGCCGCCGGTGACAAGGCAGTTTTTCCCGGTGGACGAGTCGGAA
ACCACCGTTACATCGGGAGGTGGAGCAGCACCCGATTTTCCAAGGGCCCACTGGATGGGGGTGAAATTCTGCCAG
TCCGACCCCAATACCGTTGGTGTCTTGGGGAAGTCTGCGGCGGAAGTTTCGCAGCCTCTCAAGAAGAGCCGCCGT
GGCCCGAGGTCCCGGAGCTCTCAGTACCGCGGCGTCACCTTTTACCGGCGAACTGGCCGGTGGGAATCACACATA
TGGGATTGTGGAAAACAAGTTTATTTAGGTGGATTTGATACAGCACATGCTGCAGCCCGTGCATACGATAGGGCA
GCTATTAAGTTTCGTGGAGTAGAAGCAGACATAAACTTCTGTTTGGATGATTACGAGGAAGACGTAAAACAGATG
AGAAATCTAACGAAGGAAGAATTTGTTCATGTACTTCGACGACAAAGTACTGGTTTCCCAAGAGGAAGTTCCAAG
TATAGAGGTGTTACTTTGCACAAATGTGGTAGATGGGAAGCCAGAATGGGCCAGTTCTTAGGCAAAAAGTACGTT
TATTTGGGTCTATTTGATACTGAAGTTGAAGCTGCCAGGGCTTATGATATAGCTGCCATCAAGAGTAATGGAAAG
GAAGCGGTAACCAATTTTGATCCCAGCATTTATGAAGAACTTAAAGCTCCAGATTCTTCTAGTAATGCATCTGAG
CACAATCTTGACTTGAGTTTGGGAAATTTGGCATCAAAACCAAGCAGTCGAGAATTTGGGGATAACGTTCATCAT
GCTAGAGACCAACATTCTTCATCACTGCAATTTGAACTTGAATGGCAACGTCGGGGATTGATGCCGAAGCACCAA
GTGAGTCCGATTGACAATGATTTGCAGAGAAGCGATGTATTCAATGAAACAGATACCACGCAGCTCCTAAGCCAA
ACGCACCTGTATTCTCCGGGCTCAATGAAGGTTAATGAAATGCATAGATATGGGCAATTTGTGAAAGCTAAGGAA
CCCCATATGCTTCAGATGTTTTCACCATCGTTCAGCCCACAAAATTTTCAATTTTCAAGCAGCAGCAATGGAGGC
AGTATTTGGAGCTGATGGAGTAGAGCTTTCTCTTTATATGAAAGATCATTTATCGCAAACAAATTCTCCTTGATT
ATT**AGCAGCATCATCAGGATTCC**CGTGGCTGATAATCGGCTGCAGAAATTAAACCGGCCTTCTTTCTGCTATATG
CCACTCTAAGATCTCCATTTTTTGGAGTGAGTTTTGTAAAGTTCCATGCTTTGAGCCTTTTTTGTGCTCAGTGTA
ACTAGCAGAAACCATTGGTACATGACCAGGCAGGGTTTTCAATTAGGATTGAGCACTGAGCAAAAAAACTGGAGA
AATTAATATAAAATCCCCAACTTTTCTGGAAATTAATGTTGCTCTTTTGATTAATATGTTGTTATAAGTTTAGAG
TATATAGGGGATGGAGAATGATTAATGTAACCAACTTCAAATTTATCCAACAT

>OE6A068128

Genomic: Oe6_s00162: 388220..392088 (3.87 Kb)

TAGTGGACTCTCACTCACTGGGGTGAAAATGAGGAGAGAGAGAGGATGCTTTTGGATTTGGT
TAACGTCCGAAAGTTGAAGAAGAAAGAGTTTTTCTTTTTTACAACCGATGGAAATCCCATTG
ATAACCCAAAACAAGACAAGAAGCCAACTTGTGTATCACCCCCGACCTAGGCCTACCCAACC
CTACCCTACCCTACCCCCAGAGAGGACTTGAAAAACTGGCCTCGTCTATGCTGTATTGGGCA
GATAGGGAGGATCCACGAGATCTAGTTGAATAGTTGAAATTTGGGAACAGTTTTTCTCCTTT
TTTTTGTTACAGAATCTATAGTTATATAGTTCTTTTCTTTCCTTTGAAGAGCGAACATATAC
TTCTTCTATTCTATACAAGCATTATTACAAGAGTTATGCTACTGATGGGACAGTGCTGATTA
GAATTTACGTTCTCACTCTCCTTTTTCTCTCTCTAAATTGAAGACTTTCTTACAGTATCAGA
ACTATTCAAATTTCATTGAACGGAA**ATGTGGAATCTGAACTATTCACCGGATCAAAGGATGG**
ATGAAGGTGGAAAGGGTGAACGGGTCGGATCTGAATGGAACTCGATGGATTTTTCTGAGGAA
GAAGATGCGGAGAGAAGTGGGGGTGATGGGAACAAGAAAAAAAGCAGTAAAATCTTTGGTTT
TTCTGTTTCACGTGACTGTGATGATGACGATATAGGACCATGTTCGTTGGGGAGTGAGCCGC
CGGTGACAAGGCAGTTCTTTCCTGTGGATGAGTCGGAAACCACCGTTACATCCGGCGGAGGG
GTAGCACCCAATTTTCCGAGGTCTCACTGGATGGGAGTGAAATTCTGCCTGTCCGACGCCAC
CACCGTCGGCGTCTTAGGGAAATCTGCCGCGGAAGTTTCGCAGCCACTCAAGAAGAGCCGCC
GTGGGCCAAGGTCCCGGAGCTCTCAGTACCGCGGCGTCACCTTTTACCGGCGAACCGGCCGG
TGGGAATCGCACATATGgtaaaacttatataaatcagcctttatttttttttatattatctt
cccattattcaaaagaattgatatttctgatctttttctgattcag**GGATTGTGGAAAACAA**
GTTTATTTAGgtcagtttaatttttctaacaagattatttttttatttttttgaacctgagaa
ttaatgaaatttatgttcttaatagtctgattagccaattttgaatttttccttctttcaat
tttgtatag**GTGGATTTGATACAGCACATGCCGCAGCACG**gtaagattaattcgggtttctt
acagattcttgattgagttaaagaaaaatttcaatgtgaaattcgaagagactaatgtttag
aaattaatgtgtggcag**TGCATACGATAGGGCAGCGATCAAGTTTCGGGGAGTGGAAGCAGA**
CATAAACTTTAGTTTGGATGATTACGAAGAGGACTTTAAACAGgtagtttattccaagagag
atttgggactgatggtacaatgaataagattttagcttatttttcttcttttaatacatatt
tttcctatcaaatatgctgttagagtctttgtttcacctgatcttttacaatgtgtttttgt
attatagatttagcattttgcgaattgaggtttttttggacacaagaaaggaaaaaatatcg
tctctctgaggggaaacataatattctatgttgtgtttcag**ACGGGCAATCTAACTAAGGAA**
GAATTTGTACATGTACTTCGTCGGCGAAGTACCGGATTTCCGAGAGGAAGTTCCAAGTATAG
AGGTGTTACTCTGCACAGATGTGGTAGATGGGAAGCCAGAATGGGGCAGCTCTTAGGCAAAA
Agtaatcacatgaagtgtgtgtttggggggttattcccacttttctttcaaattttcattctt
ggaagattaaatttaaacccttttgtgggttttctcttgtctaaattttgataaataatttc
cattgctcttatttgttaacag**GTATGTTTATTTGGGTCTCTTTGATACTGAAGTTGAAGCT**
GCCAGgtttgtgtgtttgagatactatctgctttaatttctgtttatttactaccctcagca
gttcaactgatagtattggatactactgaatttctggcaccaaattag**GGCTTATGATAAAG**
CTGCCATCAAGTGCAATGGAAAGGAAGCTGTTACCAATTTTGATCCCAGCATTTATGAAGAA
GAACTTGGAGATGCAGgtgaaatgaaagagcgagtagttgcgtgtgaacgttattttgtcaa
tttttttattgaattgattctggagatgggaaattttcgtgtaggacag**ATTCTTCTAGTAAT**
GCATTTGACCACAATCTCGACTTGAGTTTGGGAAATTCAGCCTCAAAGTCAATCGGTCGAGA
ATTTGGGGATGACATCCATCATACTAGAGACCAACATTCTTCATCAATGCAATTTGAAGCTG
AATGGCGATGTCAAGGATTGATACCGGAGgtatatagccataaacatcaataacttgttaat
tctatgcgataaagaatcttaaaattgtcgtttcttgattttattgtgttgattatacgtct
cgttttccag**CACCAAACGCAGCTATGTTCCACGGGCTCAGTGAAGTTTACTGAAATGCATA**
GATATGGGCCGTTTGTGAAAGCTAAAGAACCTCGTATGCTTCCGATGTTTTTACCGGCGTTC

AGCTCACAAAACTATCAAgtaagccctttaattaactacctgtgtatcagtaagcaatttt
tggcgtcagcaactttaacgtgaaaattggtgatccag**TTTCCGAGCAGCAGCGACGGTGGC**
AGGATTTGAAGCTGATGAAGGTGAGCTCCCTCTTTCGACGAAAGATCATCAATCGCAGACAA
ATTGCCCTCGATTCTTAGCAGCATCATCAGGATTCCAGCAGCTGAGAATCAGCTGCAGAAAT
TGAACTGGCTTTCTTCTTTCTGCCACACCATTTTCGAATATCTCCATTTTCGGCAAGTCTAT
AAAGTTCTATCCTTTGAGCATTTTTATTTTTTTTTGGGCTCTGTGTACCTTCCAGAGACCAT
TAGTACGTATCCATGCAGGGTTCTCAATTAGATTGAGCATtgagcaaaagaatggagaaatt
gatattctatgtttcatagttcctcattgttttgttaaaactatttacataatgtctaagga
tccgtttggtatggctttttgcatctgca

OE6A068128T1

Putative cDNA

ATGTGGAATCTGAACTATTCACCGGATCAAAGGATGGATGAAGGTGGAAAGGGTGAACGGGTCGGATCTGAATGGAACTCGATGGATTTTTCTG
AGGAAGAAGATGCGGAGAGAAGTGGGGGTGATGGGAACAAGAAAAAAAGCAGTAAAATCTTTGGTTTTTCTGTTTCACGTGACTGTGATGATGA
CGATATAGGACCATGTTCGTTGGGGAGTGAGCCGCCGGTGACAAGGCAGTTCTTTCCTGTGGATGAGTCGGAAACCACCGTTACATCCGGCGGA
GGGGTAGCACCCAATTTTCCGAGGTCTCACTGGATGGGAGTGAAATTCTGCCTGTCCGACGCCACCACCGTCGGCGTCTTAGGGAAATCTGCCG
CGGAAGTTTCGCAGCCACTCAAGAAGAGCCGCCGTGGGCCAAGGTCCCGGAGCTCTCAGTACCGCGGCGTCACCTTTTACCGGCGAACCGGCCG
GTGGGAATCGCACATATGGGATTGTGGAAAACAAGTTTATTTAGGTGGATTTGATACAGCACATGCCGCAGCACGTGCATACGATAGGGCAGCG
ATCAAGTTTCGGGGAGTGGAAGCAGACATAAACTTTAGTTTGGATGATTACGAAGAGGACTTTAAACAGACGGGCAATCTAACTAAGGAAGAAT
TTGTACATGTACTTCGTCGGCGAAGTACCGGATTTCCGAGAGGAAGTTCCAAGTATAGAGGTGTTACTCTGCACAGATGTGGTAGATGGGAAGC
CAGAATGGGGCAGCTCTTAGGCAAAAAGTATGTTTATTTGGGTCTCTTTGATACTGAAGTTGAAGCTGCCAGGGCTTATGATAAAGCTGCCATC
AAGTGCAATGGAAAGGAAGCTGTTACCAATTTTGATCCCAGCATTTATGAAGAAGAACTTGGAGATGCAGATTCTTCTAGTAATGCATTTGACC
ACAATCTCGACTTGAGTTTGGGAAATTCAGCCTCAAAGTCAATCGGTCGAGAATTTGGGGATGACATCCATCATACTAGAGACCAACATTCTTC
ATCAATGCAATTTGAAGCTGAATGGCGATGTCAAGGATTGATACCGGAGCACCAAACGCAGCTATGTTCCACGGGCTCAGTGAAGTTTACTGAA
ATGCATAGATATGGGCCGTTTGTGAAAGCTAAAGAACCTCGTATGCTTCCGATGTTTTTACCGGCGTTCAGCTCACAAAACTATCAATTTCCGA
GCAGCAGCGACGGTGGCAGGATTTGAAGCTGATGAAGGTGAGCTCCCTCTTTCGACGAAAGATCATCAATCGCAGACAAATTGCCCTCGATTCT
T**AGCAGCATCATCAGGATTCC**AGCAGCTGAGAATCAGCTGCAGAAATTGAACTGGCTTTCTTCTTTCTGCCACACCATTTTCGAATATCTCCAT
TTTCGGCAAGTCTATAAAGTTCTATCCTTTGAGCATTTTTATTTTTTTTTGGGCTCTGTGTACCTTCCAGAGACCATTAGTACGTATCCATGCA
GGGTTCTCAATTAGATTGAGCAT

OE6A079258

Genomic: Oe6_s07718:708848..710814 (- strand)

nnnnnnnnnnnnnnnnnnnnnnnnnnnnnnnnnnnnnnnnnnnnnnnnnnnnnnnnnnnn
nnnnnnnnnnnnnnntntcttgtacagtgcttttgcttagtttcaaaagttgcatttcttg
aattatgaggtgtttggacacaagaaagaaaagaaacttgatattctatgtataactattac
ag**ATGAGCAATTTAACAAAGGAAGAATTTGTGCATGTACTTCGACGACAAAGTACAGGTTTT**
CCAAAAGGGAGCTCCAAGTATAGAGGTGTTACTTTGCATAAATGTGGTAGATGGGAAGCTAG
AATGGGCCAGTTCTTAGGCAAAAAgtaaatcatctacgttacagtatgtttgtggactaatc
cctgtcctaattaccttgtttgtgctaattcatgttttattttatgaaacag**ATATGTTTA**
TCTGGGTCTTTTTGATACTGAAGTCGAGGCTGCCAGgtcagcttgtagaggagttgtctgta
tttatttctgcttaggttgccaacagggtttgtaggaaatgtattgtagaaagaattaagta
tgagtataatacttttccggggcttaattacaatgtctgggaattatag**GGCTTATGATAAA**
GCTGCCATCAAGTGTAATGGAAAGGATGCTGTTACAAATTTTGATGCCAGCATTTATGATGA
AGAACTCAGAGGTGCAGgtaaaattaatggtatttgtcacgtttgttccggcatagttctta
aaattctttgttcaactgatgtagagacgaaaattcatttatttattgaatttttttattt
ttttttttttgggggttgtactacag**AATCTTCTAACAATGCAGAATATCATAATCTAGAC**
TTGAGTTTGGGGAATGCAGCTTCAAAGTGTAGCAGCAGAGAATTGGAAGATGAAAATCATGA
TCAGCATTGTTCGACAATGCCGTTTGAATTTGATTTGCGACTCCAGGGATCAAGATCCGAGg
tatatagacattaatgtgccttgatttgatgccaaaatccaaaatttgcagtgtttttatt
tgatgacttttatgaatcacag**CTTAATTTCCAGAATGGACTTGGTCAAATTGACAGAAGGG**
ATGGATATAATGAAAATGATACTTTACAGCTTCTAAGCCAAACACACCTGCACTCTCCCGGT
TCATTGAAGGTTAACGAATTGCACCAATATAGACAGTTCATGGAAGGCAATGAGCCAAGAAT
GCTAATGTTCTCTTCACCGGTAGGCTCACAAAGTTATCGAgtaagtgcttaaagttcaataa
ccatacacgagtatatacaaaattcttaacgagtgttggaactgaacatgacaattgacag**A**
ATCAGTTACCGAGCTGCAGCAATGGTGGTGGAATTAGAGGCAACGGAGAAGTGATTTCTATC
TCAACAAATGATCAGCTATGGCAAACAAAATCTCCTCAATTATTTGCTACTGCTGCAGCATC
ATCAGGATTCGGACAGCAAATACTGAGACCCCAAAATTGGCTGCACAAAAGTATTGTCCGCC
CACTCACGAGACCCTCATAGCTGACCTAATCTTGTGTTCCGCTTAGTCGTCGTCTAGGATTC
TGTCATTAAAGATCTCCATTTTCTCTGAATCTGTAAAATTCCTAATAAGGGACAAAAATTTA
TGATTAGAATCTGAAAAGGGGGATTGGTAATTTGTCCAAACTAACGGGAAAAAAGAAATCTT
GGGTTGGATCTTCTGCATTGCTGATTAGTAACTGGTGATACAGTATGATTGTAATATCTATA
CAAGTTTTCTGGATCTTTTGTGAAAAAATTCCAATGTACAAGATATGGTATTAGTTCTTATC
ATAGTATTATTAGACCTTAAA

OE6A079258T4

Putative cDNA

ATGAGCAATTTAACAAAGGAAGAATTTGTGCATGTACTTCGACGACAAAGTACAGGTTTTCCAAAAGGGAGCTCCAAGTATAGAGGTGTTACTTTGCATAAATGTGG
TAGATGGGAAGCTAGAATGGGCCAGTTCTTAGGCAAAAAATATGTTTATCTGGGTCTTTTTGATACTGAAGTCGAGGCTGCCAGGGCTTATGATAAAGCTGCCATCA
AGTGTAATGGAAAGGATGCTGTTACAAATTTTGATGCCAGCATTTATGATGAAGAACTCAGAGGTGCAGAATCTTCTAACAATGCAGAATATCATAATCTAGACTTG
AGTTTGGGGAATGCAGCTTCAAAGTGTAGCAGCAGAGAATTGGAAGATGAAAATCATGATCAGCATTGTTCGACAATGCCGTTTGAATTTGATTTGCGACTCCAGGG
ATCAAGATCCGAGCTTAATTTCCAGAATGGACTTGGTCAAATTGACAGAAGGGATGGATATAATGAAAATGATACTTTACAGCTTCTAAGCCAAACACACCTGCACT
CTCCCGGTTCATTGAAGGTTAACGAATTGCACCAATATAGACAGTTCATGGAAGGCAATGAGCCAAGAATGCTAATGTTCTCTTCACCGGTAGGCTCACAAAGTTAT
CGAAATCAGTTACCGAGCTGCAGCAATGGTGGTGGAATTAGAGGCAACGGAGAAGTGATTTCTATCTCAACAAATGATCAGCTATGGCAAACAAAATCTCCTCAATT
ATTTGCTACTGCTGCAGCATCATCAGGATTCGGACAGCAAATACTGAGACCCCAAAATTGGCTGCACAAAAGTATTGTCCGCCCACTCACGAGACCCTCATAGCTGA
CCTAATCTTGTGTTCCGCTTAGTCGTCGTCTAGGATTCTGTCATTAAAGATCTCCATTTTCTCTGAATCTGTAAAATTCCTAATAAGGACAAAAATTTATGATTAG
AATCTGAAAAGGGGGATTGGTAATTTGTCCAAACTAACGGGAAAAAAGAAATCTTGGGTTGGATCTTCTGCATTGCTGATTAGTAACTGGTGATACAGTATGATTGT
AATATCTATACAAGTTTTCTGGATCTTTTGTGAAAAAATTCCAATGTACAAGATATGGTATTAGTTCTTATCATAGTATTATTAGACCTTAAA

OE6A061030

Genomic: Oe6_s06094:146226..149985

aggaaaaagaacgcttaatatgccagacatatataagtagaaatatatttatataaaatcac
cccgtcaacagagccctctcctctacctgtatatgaatttcacacacacatggagatagaga
gagagagagagagagagagagagagagaagtaagagttcttggattctagagagagagagac
actgcttttggtcaaatcttgaaaaactggcctcgtctatgccgtactgggcagccatagga
aacatgagagatctagttgaatatttgaaactggaataaacttttcttttacaactctttaa
ggtaataagtttttatgttgtttcaagaagtcacgtataccagttctatttgagcattattt
tatattggtagccaaaaggaatatctataatctatacccactcaaccctcaatattctactc
ctacaaactcaaaaacgagcttttgtagcctcccaa**AATTTGGCTTCTGATGAGACAGTACT**
GATCAGAATTCTGAAAACCCATCAACTATCTTCCTCTCTAATCTTTCTTTAACCGACCATCT
CTTTTTGGTTTTCTATCTTTTTTCCTCTTTGGGGTTAGTTTTCAGTTTTTCATCGGTAGAGA
AATGTGGAATCTCAATGATTCACCAGATCAAACTAGGGGGGATGAATCCGAGGGTTATTCGG
ATGACAAGCTTAAAGGGGTCGGATCTGTGTCTAATTCAAGCTCGTCGGCGGTAGTTATCGAG
GATGCTTCAGAAGATGAAGATGCAGAAAGAGGTAGAAAGAAAAGGAGCAGCAAAATATTTGG
TTTTTCTTTTGCTCATAACGATGATGACACGGATCCATGCTCGTCGCCGGTTACCCGGCAGT
TCTTTCCGGTCGATGAGTCTGAAACTGGGGCTACATCAAGCGACCGACCACTCAATTTACCG
AGAGCTCAATGGTTTGGAGTTAAATTCTGTAAGTCTGAATCCCAAGGCGGCGGTGGAGGTGG
TTTTGGGAAGTCTGCAGCGGCTGAGGTTGCACAGCCACTCAAGAAAAGCCGCCGGGGGCCTA
GGTCTCGTAGCTCTCAATATCGTGGGGTTACCTTTTATCGGAGGACTGGCCGGTGGGAGTCA
CACATATGgtatacatttacaaatcaaaaactttatctttgcttttcctttaatgctagttt
tcactttgtcccaacaaaattgtttgttccattcaaatacaattcatctgtgtttcttgttt
ttacttttctttccgattcag**GGATTGTGGAAAACAAGTTTATTTGG**gttagtatttccttc
tttattctccatatttagtaaacaagtttattcttttctcaacttgagcatcaagttcttgc
tttatcatctaatttgtcaaatgggcttttgtgttggaaatttatag**GTGGATTTGATACAG**
CACACGCGGCAGCTCGgttagcttttcaccttctctaactcttatttccaattccacatta
tttacctataatccgaagaaactaatgttcagacatgaatctattacag**TGCATATGATAGG**
GCAGCCATTAAGTTCCGTGGAATGGAAGCAGACATAAACTTTAATCTAGAAGATTACGAAGA
AGACCTAAAACAGgtacttcatttgaagagagaggggggaggggggcttctttatcatttc
acctaatcttgtacagtgccttttccttagtttcaaattcatgaattattaggtgattagta
caagaaagaaatcaaaaatgatattctatgttgaaatatttcag**ACGAGCAATTTAACCAAG**
GAAGAATTTGTGCATGTACTTCGTCGACAAAGTACTGGTTTTCCAAGAGGGAGCTCCAAGTA
TAGAGGTGTTACCTTGCACAAATGTGGTAGATGGGAAGCTAGAATGGGCCAGTTCTTGGGCA
AAAAAgtaaatcacatattacagtatccttgtggactaatccttgtcttaattaccttgttt
tatgctaatgcatgttttattttatgaaacag**ATATGTTTATTTGGGTCTGTTTGATACTGA**
AGTCGAAGCTGCCAGgtcagtttgtagagtagttgactgtatttatttctgcttagtgttgc
cagcagggtttgcaggattgagtaggagtataatactttctggggcttaattatattgtctg
ggaattatag**GGCTTATGATAAAGCTGCCATCAAGTGTAATGGGAAGGATGCTGTTACAAAT**
TTTGATCCCAGTATTTATGATGAAGAACTCAAAGGTGCAGgtaaaattaatgaggacttagt
cgcgttcgaaggagttgaaaattcttcgttgaactgattttggagatgaaattttacttgtc
tgatttgaagtttttggtgtgtgacag**AATCTTCTAACAACACAGCAGATCACAATCTAGAC**
TTGAGTTTGGGGAATTTAGCCTCAAGGCGTAGCAGCAAAGAAATGGAAGACAATAGTCGAGA
TCAGCATTGTTCGACAATGCCATTTGAATTTGATTGGCGACACCAGGGGTCGCGATCAGAGg
tatatagacaccaatgtgcctttactttgatgccaaaacttcagtgttatatatgtgatgac
ttttgtgaaacacag**CTTAGTTTCCAGAATCAACCGAGCCCAGTTGACAAAAGAGACCGAAG**
AGATGGATATAATGAAACCGATACTTTACGGCTTCTAAGCCAAACGCACATTCACTCTCCCA

GTTCGTTGACGGCTAACGAAATGCACCAATATGGACAGTTCACGAAAGGCCACGATCCCCGC
TTTCTTCAAATGTTTGCACCATTTGGTTCCCAAAATTTTCAAgtaagtgcttaaagatcgat
atccatacgtgtaaattaacgctcaaaagtaacgaatgctgaactgaacatagaaatcgaca
g**ATTCAGTATCCGAGCAGCAGCAATGTTGGTGGTGGAACTACGGGCAATGGAGGAGAAA**
TTTCTCCCTCAACAAATGATCAGCAGTGGCAGACATATTCACCTCAATTATTTGCTACTGCT
GCAGCATCATCAGGATTCCGACAGCAAATACTGAAACCTCAAAATTGGCTGCAGAAAAATAG
TGTCCACCCAATTACGAGACCCTCATAGTCGGCATATTCTCGTTTTCTGGCAGGTCATTGTC
TTCTAATTCTGCCATCAAAAGATCTCCATTTTCTTTGAAGTCTGTAAAAATCCTAATCAGGG
ACAAAAAAAAATTATGATTAGAATCTGAAACGAGGTGGAGAAATTTGGAAAAATCAAATGTT
AATTGGTCCAAACTAATGGCAAAAAATCTTGGATAAGATCTTCTGCCTTGTTTTCAACGAAC
AGTTTTCAATGCGTTGCGAAAATTCTTTGATGGCCTGATTTGGTGGCACTCTATATACCTCC
Attttagataccccctcgactagaaggcttggactaccgtaacgactctaattctagagttcg
actcatggttgtatagctatttcaacgcgataagtttttccaatatgcttgctaataatgca
cccttttagatgtgatcttcattacaactgagggatttggaaggagtaatgctatttgtagt
ccttcatcgtcgtagtttttatgtggtatatatgtggtggatagtgatttgatatatgact
aatacattaatataacaaataaaaaatcgtcatataggatatcacgtaaagtgactacaaag
attgtaatgagcgattatgaatattatttactcatttggaa

OE6A061030T2

Putative cDNA

AATTTGGCTTCTGATGAGACAGTACTGATCAGAATTCTGAAAACCCATCAACTATCTTCCTCTCTAATCTTTCTTTAACCGACCATCTCTTTTT
GGTTTTCTATCTTTTTTCCTCTTTGGGGTTAGTTTTCAGTTTTTCATCGGTAGAGAA**ATG**TGGAATCTCAATGATTCACCAGATCAAACTAGGG
GGGATGAATCCGAGGGTTATTCGGATGACAAGCTTAAAGGGGTCGGATCTGTGTCTAATTCAAGCTCGTCGGCGGTAGTTATCGAGGATGCTTC
AGAAGATGAAGATGCAGAAAGAGGTAGAAAGAAAAGGAGCAGCAAAATATTTGGTTTTTCTTTTGCTCATAACGATGATGACACGGATCCATGC
TCGTCGCCGGTTACCCGGCAGTTCTTTCCGGTCGATGAGTCTGAAACTGGGGCTACATCAAGCGACCGACCACTCAATTTACCGAGAGCTCAAT
GGTTTGGAGTTAAATTCTGTAAGTCTGAATCCCAAGGCGGCGGTGGAGGTGGTTTTGGGAAGTCTGCAGCGGCTGAGGTTGCACAGCCACTCAA
GAAAAGCCGCCGGGGGCCTAGGTCTCGTAGCTCTCAATATCGTGGGGTTACCTTTTATCGGAGGACTGGCCGGTGGGAGTCACACATATGGGAT
TGTGGAAAACAAGTTTATTTGGGTGGATTTGATACAGCACACGCGGCAGCTCGTGCATATGATAGGGCAGCCATTAAGTTCCGTGGAATGGAAG
CAGACATAAACTTTAATCTAGAAGATTACGAAGAAGACCTAAAACAGACGAGCAATTTAACCAAGGAAGAATTTGTGCATGTACTTCGTCGACA
AAGTACTGGTTTTCCAAGAGGGAGCTCCAAGTATAGAGGTGTTACCTTGCACAAATGTGGTAGATGGGAAGCTAGAATGGGCCAGTTCTTGGGC
AAAAAATATGTTTATTTGGGTCTGTTTGATACTGAAGTCGAAGCTGCCAGGGCTTATGATAAAGCTGCCATCAAGTGTAATGGGAAGGATGCTG
TTACAAATTTTGATCCCAGTATTTATGATGAAGAACTCAAAGGTGCAGAATCTTCTAACAACACAGCAGATCACAATCTAGACTTGAGTTTGGG
GAATTTAGCCTCAAGGCGTAGCAGCAAAGAAATGGAAGACAATAGTCGAGATCAGCATTGTTCGACAATGCCATTTGAATTTGATTGGCGACAC
CAGGGGTCGCGATCAGAGCTTAGTTTCCAGAATCAACCGAGCCCAGTTGACAAAAGAGACCGAAGAGATGGATATAATGAAACCGATACTTTAC
GGCTTCTAAGCCAAACGCACATTCACTCTCCCAGTTCGTTGACGGCTAACGAAATGCACCAATATGGACAGTTCACGAAAGGCCACGATCCCCG
CTTTCTTCAAATGTTTGCACCATTTGGTTCCCAAAATTTTCAAATTCAGTATCCGAGCAGCAGCAATGTTGGTGGTGGAACTACGGGCAAT
GGAGGAGAAATTTCTCCCTCAACAAATGATCAGCAGTGGCAGACATATTCACCTCAATTATTTGCTACTGCTGCAGCATCATCAGGATTCCGAC
AGCAAATACTGAAACCTCAAAATTGGCTGCAGAAAAATAGTGTCCACCCAATTACGAGACCCTCA**TAG**TCGGCATATTCTCGTTTTCTGGCAGG
TCATTGTCTTCTAATTCTGCCATCAAAAGATCTCCATTTTCTTTGAAGTCTGTAAAAATCCTAATCAGGGACAAAAAAAAATTATGATTAGAAT
CTGAAACGAGGTGGAGAAATTTGGAAAAATCAAATGTTAATTGGTCCAAACTAATGGCAAAAAATCTTGGATAAGATCTTCTGCCTTGTTTTCA
ACGAACAGTTTTCAATGCGTTGCGAAAATTCTTTGATGGCCTGATTTGGTGGCACTCTATATACCTCCA

Group AP2_1 genes

>OE6A037406T2

Putative cDNA

ATGTTGGACCTTAATCTGACTGTTGATGCGAATGGATCGATCTTCAATGGAACTGAAACAGT
TTCGGAGAAGCAACTCGAATTGGGCGGAGGACAAATAGAGAATTCTGGTAGTTCTGTTTCCT
CTGTTGTCAATGTGGAAATCTCCAACGCTGTGGGCGACGAGGACTCCTGTTCGAATCCTTTC
TTCAGTTCCGATTTATTGAAGAGTAATGGCGAAGTGAATCACTATGAGGACAGTGAGAACCG
TAGCGGTAATTTCGTGACCAAGGAGTTTTTCCTGTGAGCGAGGGAGCTGCTGTGGCGCAGC
AGTGTCAGAGGTTGGATCTTTCGAGGAATTACAGCGGAATGGTGGAGCAGAGGATTGTCGCT
CAGCCGCAGCAGAGACAGCAGGTGAAGAAGAGCCGGAGAGGACCGAGGTCCCGCAGCTCTCA
GTACCGTGGAGTCACATTCTATCGTAGAACTGGACGATGGGAATCTCACATATGGGACTGTG
GAAAACAAGTTTATTTAGGGGGCTTCGACACTGCACACGCTGCGGCTAGGGCGTATGATCGT
GCTGCGATTAAGTTCCGTGGGCTTGATGCTGATATCAATTTCAGCGTAGTTGATTATGAAAA
AGAACTTAAGCAGATGAAGAATTTAACAAAGGAAGAATTTGTACACATACTGCGTCGCCAGA
GCACTGGGTTTTCTAGAGGAAGTTCGAAGTACAGGGGAGTCACGTTGCACAAATGTGGCCGT
TGGGAAGCTCGAATGGGACAACTTCTTGGCAAAAAGTATATCTATCTTGGATTGTTTGACAG
TGAGATAGAAGCTGCAAGGGCATATGACAAGGCAGCTATAAAATGTAATCGAAGGGAAGCAG
TCACTAATTTTGATCCAAGCACATATGAAGAGGAGATTAGCTCTGAGGCTGCTGATGGAAAC
CATAATCTAGATCTGAACTTGGGCATTGCTCCCCCTTGTTCTGCGAGTGGCCAAAATAGGAC
CATTGGTGCTGATGGCTTCCAAATTCACTGCACCTCATATAGCACCCTGCCAGAAAATGAAG
AAAGGATGCATAACTCTGCTTCAGCCACAATGGAAACAATATTGTCACATGGACATGTAGAT
GTGCTTGACCATCCTCGCTTCAGGAAGGGAGTGAATTCCAGTTCTTGTCCCATTTATAAGGG
ATCAGAAATAGGGAAGAGTAGGGAACTTAATTTTTCCGCAAATTGGGCATGGCAAACACAGA
ACCCCATTAATAGGGTTCCTGCTGATCCACTCTTCTCTGCTGCAGCATCATCAGGATTCGCT
AATTCAAGTATTGCTGCTTCCTCAGCTGCTGTTCTTCCAAGTGAAACAATATCCCAACGCCA
TTATCTACCTTCAGTCATCAATTCACTTTATTACGGCAGGAGCTGA

>OE6A055418T1

Putative cDNA

ATGTTGGACCTTAATCTGACTATTGATGCGAATGGATCGATCTTCAATGGAATTGAAACAAT
TTGGGAAAAGCAACACGAATTTGGCGGAAGGCAAATGGAGAATTCCGGCAGTTCTAGTTCTT
CTGTTGTTAATATGGAAATCTCCAACGCCGTCGGTGATGAAGACTCCTCTTCGAATCCTTTC
TTCAGTTTCGATATATTGAAAAGTAATGGAGAAGTGAACGACTATAGGGAGAGTGAGAATCA
AAGCCACGGATTTGTGACGACGGAGTTTTTCCCGGGAGCGGCTGTGGCGAAGCAGTGTCAAT
GGTTGGATCTTTCGGGGAATTACGGTGGGGCGGTGGAGCAGAGGATCGTCGCTCAGCAGCAG
CCGCAGCAGAGACAGCAGGCGAAGAAGAGCAGGAGAGGACCAAGGTCTCGAAGCTCTCAGTA
CCGTGGAGTTACATTCTATCGTAGAACTGGACGATGGGAATCTCACATTTGGGACAGTGGAA
AACAAGTTTATTTAGGGGGCTTCGACACTGCACATGATGCAGCTAGGGCATATGATCGTGCT
GCGATCAAATTCCGTGGGATTGATGCTGATATCAATTTCAACGTAGTTGATTATGAAAAAGA
CCTTAAGCAGATAAAAAATTTAACCAAAGAAGAATTTGTACATATACTGCGTCGCCAGAGCA
CTGGGTTTTCTAGAGGAAGTTCGAAGTACAGGGGAGTCACATTGCACAAATGTGGCCGTTGG

GAAGCTCGAATGGGACAATTTCTTGGCAAAAAGTATATCTATCTCGGGTTGTTTGACAGTGA
GTTGGAAGCTGCGAGGGCATATGACAAAGCTGCTATAAAATGTAACGGAAGGGAAGCAGTCA
CTAATTTTGAGCCAAGCACATACGAAGAGAAGATTAGCTCTGAGGCAGCTGGTGGAAACCAT
AATCTTGATCTGAACTTGGGCATTGCTCCCCCTTGTTCAACTGATGGCCAAATCAGGACAAT
TGATGCTGATGGCTTCCAGATTCACTGCAGCTCAGATGGCACCCTTCCTGTAAATGGAGCAA
GGGTGCATAAATATGCTTCAGCCACAATGGACACACAATTGTCAAATGGATATATCGATGTG
CTTGACCATCCTCACATCTGGAAAGGTGTGAATTCCAGTTCTTGTCCCATTTATAAGGGATC
AGCAATAGAGAAGAGTATGGACTTTCATTATTCCGCTGATTGGGCATGGCAAACACAGAACC
CTATTAATGGGGTTCCCACAGAGCCACTCTTCTCTACTGCAGCATCATCAGGATTCGCTAAT
TCAAGTATTGCTGCTTCCTCAGCTGCTGTTCATACAAGTGAAACTGTATCCCGACACCATTG
TCTACCTTCTGTCACCAATTCACCTTACTATGGCATGAGCTGAAATAACAGGCAGTTAGATT
ACGACACCATCTAGACTTCTGTACCTATAAAACAGGTCGTTTGCAACATAACAATATAGGGT
TCTAATTGATTCTGAGTTTCTTTTTTGCTGATCTTTGTTTTCGTCGTCCTGAAAACTTTAGT
GAATGATGCTTATGTGCACAAATGTTGATTGTAGAAACTGGA

>OE6A105872T1

Putative cDNA

CTTACAGTCCCCCTATGTGGCTATGTTTTTCAACAATACGTACATTTTTTCTACCGTGCAAC
TATATATAGTAGTGAGGGAAATTGGAAATTTGAGGTGAATTTTTTAGCATAGAAGAGTGATG
TTGGACCTTAATCTGACTATTGATGCGAATGGATCGATCTTCAATGGAATTGAAACAATTTG
GGAAAAGCAACACGAATTTGGCGGAAGACAAATGGAGAATTCCGGCAGTTCTAGTTCTTCTG
TTGTTAATATGGAAATCTCCAACGCCGTCGGTGATGAAGACTCCTCTTCGAAGCCTTTCTTC
AGTTTCGAAATATTGAAAAGTAATGGAGAAGTGAACGACTATAGGGAGAGTGAGAATCAAAG
CCACGGATTTGTGACGACGGAGTTTTTCCCGGGAGCGGCTGTGGCGAAGCAGTGTCAATGGT
TGGATCTTTCGGGGAATTACGGTGGGGCGGTGGAGCAGAGGATCGTCGCTCAGCAGCAGCCG
CTGCAGAGACAGCAGGCGAAGAAGAGCAGGAGAGGACCAAGGTCTCGAAGCTCTCAGTACCG
TGGAGTTACATTCTATCGTAGAACTGGACGATGGGAATCTCACATTTGGGACAGTGGAAAAC
AAGTTTATTTAGGGGGCTTCGACACTGCACATGATGCAGCTAGGGCATATGATCGTGCTGCG
ATCAAATTCCGTGGGATTGATGCTGATATCAATTTCAACGTAGTTGATTATGAAAAAGACCT
TAAGCAGATAAAAAATTTAACCAAAGAAGAATTTGTACATATACTGCGTCGCCAGAGCACTG
GGTTTTCTAGAGGAAGTTCGAAGTACAGGGGAGTCACATTGCACAAATGTGGCCGTTGGGAA
GCTCGAATGGGACAATTTCTTGGCAAAAAGTATATCTATCTCGGGTTGTTTGACAGTGAGTT
GGAAGCTGCGAGGGCATATGACAAAGCTGCTATAAAATGTAACGGAAGGGAAGCAGTCACTA
ATTTTGAGCCAAGCACATACGAAGAGAAGATTAGCTCTGAGGCAGGTAATGGAGCTGGTGGA
AACCATAATCTTGATCTGAACTTGGGCATTGCTCCCCCTTGTTCTACTGATGGCCAAATCAG
GACAATTGATGCTGATGGCTTCCAGATTCACTGCAGCTCAGATGGCACCCTTCCTGTAAATG
GAGCAAGGGTGCATAAATATGCTTCAGCCACAATGGACACACAATTGTCGAATGGATATATC
GATGTGCTCGACCATCCTCACATCTGGAAAGGTGTGAATTCCAGTTCTTGTCCCATTTATAA
GGGATCAGCAATAGAGAAGAGTATGGACTTTCATTATTCCGCTGATTGGGCATGGCAAACAC
AGAACCCTACTAATGGGGTTCCCACAGAGCCACTCTTCTCTACTGCAGCATCATCAGGATTC
GCTAATTCAAGTATTGCTGCTTCCTCAGCTGCTGTTCATACAAGTGAAACTGTATCCCGACA
CCATTGTCTACCTTCTGTCGCCAATTCACCTTACTATGGCATGAGCTGA

>OE6A031451T2

Putative cDNA

GTATTATACACTCACACGCCATAAACGTTTTCTCTTTTTCTCTCTCTAGATAACTCTTCCAA
TTTTCTCTCTCTAGGTTTAGATCCGAGAGTTATTTTTATATATAGCTCTCCATGGACTTGCT
CCTTTTCTGCTAATACGAGAGAGAAAGCAATGCAAATTATTAATCCTGTTGTAACTTCTGAC
ATATACATACATCAATAGATACAACCTTTACGTGTTTCTATATATGTATAGACATTCTTTTC
TTCTTCACCATTTCTTCCCTCTTTCTATTTCTCATTTCCTATCTCTATCTAGATTATGATTC
AAGCTGATTGATGGTATATATGAACAGATAGGTGCACAGAGTAGTGTGAAAATAATTGGAAA
ATCAGGTTGATATTCGAGGAGCTGAGTGATGTTGGACCTTAATTTGACTAGCGATGCTAACG
AATCGATCACAAATTTAGGCGAAATGGTGTTGGAAAAGCATCTAGATGGCTCCGGGGGTCAG
ATGGAGAATGCCGGCAGTTATAATTCCTCTGTTGCCAACGTGGATACCACAAGCAGTGCCGG
TGACGGGGACTCTTGTGATAACCAATTCATCGGTTTCGATATATGGAAGGGGAATGGTGATT
ATGAAGAGAGTGAGTATCAGAGTGGCGTTTCTGTGACGAAGGAGTTCTTCCCTGTGACCGCC
GAAGGGACATCGGCGGCACGGCATTGTCAATGGTCCGACGTTTCAGGGAATTATGGTGGAGT
GGCGGCTCAAAGGACAATATCTCAGCAGAAGCAGCAGATTAAGAAGAGTCGTAGAGGACCGA
GGTCTCGCAGCTCTCAATACCGTGGAGTCACATTTTATCGCAGGACCGGTAGATGGGAGTCT
CACATTTGGGATTCTGGAAAACAAGTTTATTTGGGAGGATTTGACACTGCACATGCTGCAGC
TAAGGCATATGATCGTGCTGCAATAAAGTTTCGTGGAATTAATGCTGATATAAATTTCAACG
TCAGTGATTATGAAGAAGAACTTAGCCAGATTATGAATTTGCCCAAGGAAGAGTTTGTGCAG
ACACTTCGTCGCCAGAGTACGGGCTTCTCTAGAGGAAGTTCAAAGTACAGGGGGGTTACATT
ACACAAATGTGGGCGATGGGAAGCTCGAATGGGCCAGCTTCTTGGAAAGAAGTATATCTATC
TTGGATTGTTTGACAGCGAAATAGAAGCTGCAAGGGCATATGATAAGGCTGCTATAAAATCC
AATGGAAAGGAAGCTGTCACCAATTTTGAGCCTAGCACATACGAAGAAGGGATAAGCTCTGA
AGCTGAAAATGGAGGCATGAGCCATAATTTGGATCTTAACTTGGGGATTGCTCCTCCTCATC
TTGCTGATGGCCAAAATATGAGCATTGATGCTAAAAGCTTTCAGCTCCACAGTGGCTCAGAG
GGCCTGCCTGAAAATAGAGCATGGTTTTCTCACAAATGCAGCACTCTGCTTCAACCTCAGAC
GGAACTGTATTACTGCGTGAACATGTAAATGTCGGTGGATCCCCTTACCTCTGGAATGGATT
GAATTCCGGTTTTTATCCCATTTATAAGGGAAAGGCAATAGGGAAGAGTATGGCAGTCAATT
CATCAGCAAACTGGGCATTGCCGATGCAAAGCCCCTTCAATGGGGTTCCTCCACGGCTACTC
TTTTCTACTGCAGCATCATCAGGATTCGCTAATTCAACTGTCTCTACTTCCTCCGCTGCTGT
TGTGCAAATCTCGAATACAATAAAATCCCCACACTTTTATCTGCCGTAAATGGCCAATATAG
AGAACACGGCTCATTATACTCTTTTCATTCGGCATAACCAGACATTTTATTTTGTAAAACAT
TCTAAAATCTCTACCGTATAATAGCACGGCTTGCTACTCTCTGTGCATATGGTTTCTCTATC
ATCACTGGGATAAGCTTTCCTTTTTTGCAATTCCTGTAATGAGGTTTGCAATATTTGAACAA
CTTGTTACTTAAGAGGATATGTTGAGAGCTAAATGTCATATTTTTTCTGTAAATTTTATAAA
AGTTGATGAATTTTGGGGTTGAAAGTTGATAATAATTACGAGTCGCATCAAATGTG

Protein Sequences used in Alignment

>SMZ

MLDLNLKIFSSYNEDQDRKVPLMISTTGEEESNSSSSSTTDSAARDAFIAFGILKRDDDLVP
PPPPPPHKETGDLFPVVADARRNIEFSVEDSHWLNLSSLQRNTQKMVKKSRRGPRSRSSQYR
GVTFYRRTGRWESHIWDCGKQVYLGGFDTAYAAARAYDRAAIKFRGLDADINFVVDDYRHDI
DKMKNLNKVEFVQTLRRESASFGRGSSKYKGLALQKCTQFKTHDQIHLFQNRGWDAAAIKYN
ELGKGEGAMKFGAHIKGNGHNDLELSLGISSSSESIKLTTGDYYKGINRSTMGLYGKQSSIF
LPMATMKPLKTVAASSGFPFISMTSSSSSMSNCFDP

>SNZ

MLDLNLGILSTHNEDEDCKVPTSIFIQEEDSINPSNDNLSLITFGILKRNVEILPPPPPPPP
PPPPSENELSGPGNEWLDLSSMQRNKQETLVMKKKSRRGPRSRSSHYRGVTFYRRTGRWESH
IWDCGKQVYLGGFDTAYTAARAYDRAAIRFRGLQADINFIVDDYKQDIEKMKNLSKEEFVQS
LRRASASLARGGSKYKNTHMRNDHIHLFQNRGLNAAAAKCNEIRKMEGDIKLGAHSKGNEHN
DLELSLGISSSSKVRILEPADYYMGLNRSVTSLHGKPLPGYLPITEIKPLKTVVASSGFPFI
TMINPSSLSLSCFDP

>TOE2

MLDLNLDVDSTESTQNERDSITVKGVSLNQMDESVTSNSSVVNAEASSCIDGEDELCSTRTV
KFQFEILKGGGEEEEEDDDERSAVMMTKEFFPVAKGMNFMDSSAQSSRSTVDISFQRGKQGG
DFIGSGSGGGDASRVMQPPSQPVKKSRRGPRSKSSQYRGVTFYRRTGRWESHIWDCGKQVYL
GGFDTAHAAARAYDRAAVKFRGLEADINFVIGDYEEDLKQMANLSKEEVVQVLRRQSSGFSR
NNSRYQGVALQKIGGWGAQMEQLHGNMGCDKAAVQWKGREAASLIEPHASRMIPEAANVKLD
LNLGISLSLGDGPKQKDRALRLHHVPNNSVCGRNTMVQFFLQCTKYDLLYFHMRFAATMENH
MAAAACDTPFNFLKRGSDHLNNRHALPSAFFSPMERTPEKGLMLRSHQSFPARTWQGHDQSS
GGTAVAATAPPLFSNAASSGFSLSATRPPSSTAIHHPSQPFVNLNQPGLYVIHPSDYISQHQ
HNLMNRPQPPP

>TOE1

MLDLNLNADSPESTQYGGDSYLDRQTSDNSAGNRVEESGTSTSSVINADGDEDSCSTRAFTL
SFDILKVGSSSGGDESPAASASVTKEFFPVSGDCGHLRDVEGSSSSRNWIDLSFDRIGDGET
KLVTPVPTPAPVPAQVKKSRRGPRSRSSQYRGVTFYRRTGRWESHIWDCGKQVYLGGFDTAH
AAARAYDRAAIKFRGVDADINFTLGDYEEDMKQVQNLSKEEFVHILRRQSTGFSRGSSKYRG
VTLHKCGRWEARMGQFLGKKYIYLGLFDSEVEAARAYDKAAINTNGREAVTNFEMSSYQNEI
NSESNNSEIDLNLGISLSTGNAPKQNGRLFHFPSNTYETQRGVSLRIDNEYMGKPVNTPLPY
GSSDHRLYWNGACPSYNNPAEGRATEKRSEAEGMMSNWGWQRPGQTSAVRPQPPGPQPPPLF
SVAAASSGFSHFRPQPPNDNATRGYFYPHP

>AP2

MLDLNLNADSPESTQYGGDSYLDRQTSDNSAGNRVEESGTSTSSVINADGDEDSCSTRAFTL
SFDILKVGSSSGGDESPAASASVTKEFFPVSGDCGHLRDVEGSSSSRNWIDLSFDRIGDGET
KLVTPVPTPAPVPAQVKKSRRGPRSRSSQYRGVTFYRRTGRWESHIWDCGKQVYLGGFDTAH
AAARAYDRAAIKFRGVDADINFTLGDYEEDMKQVQNLSKEEFVHILRRQSTGFSRGSSKYRG
VTLHKCGRWEARMGQFLGKKYIYLGLFDSEVEAARAYDKAAINTNGREAVTNFEMSSYQNEI
NSESNNSEIDLNLGISLSTGNAPKQNGRLFHFPSNTYETQRGVSLRIDNEYMGKPVNTPLPY

GSSDHRLYWNGACPSYNNPAEGRATEKRSEAEGMMSNWGWQRPGQTSAVRPQPPGPQPPPLF
SVAAASSGFSHFRPQPPNDNATRGYFYPHP

>TOE3

MWNLNDSPDHHEESDSRGNPVGHVSNGMSQSATWLPFVLPVTRNFFPAQSMEPGVRWSGFNS
VGKSDPSGSGRPEEPEISPPIKKSRRGPRSRSSQYRGVTFYRRTGRWESHIWDCGKQVYLGG
FDTAHAAARAYDRAAIKFRGVDADINFDIEDYLDDLKQMGNLTKEEFMHVLRRQSTGFPRGS
SKYRGVTLHKCGRWESRLGQFLNKKYVYLGLFDTEIEAARAYDKAAIKCNGKDAVTNFDPKV
YEEEEDLSSETTRNGHNLGLSLGESSSEEFRLKSDIASIRSRIRDEERLLGSDLSLAMMTTT
VRSEKQQSDGGGNRVVGMAASSGFSPQPSPYRIPRTFHFSRP

>OE6A061030P1
MWNLNDSPDQTRGDESEGYSDDKLKGVGSVSNSSSSAVVIEDASEDEDAERGRKKRSSKI
FGFSFAHNDDDTDPCSSPVTRQFFPVDESETGATSSDRPLNLPRAQWFGVKFCKSESQGG
GGGGFGKSAAAEVAQPLKKSRRGPRSRSSQYRGVTFYRRTGRWESHIWDCGKQVYLGGFD
TAHAAARAYDRAAIKFRGMEADINFNLEDYEEDLKQTSNLTKEEFVHVLRRQSTGFPRGS
SKYRGVTLHKCGRWEARMGQFLGKKYVYLGLFDTEVEAARAYDKAAIKCNGKDAVTNFDP
SIYDEELKGAESSNNTADHNLDLSLGNLASRRSSKEMEDNSRDQHCSTMPFEFDWRHQGS
RSEVYRHQCAFTLMPKLQCYICDDFCETQLSFQNQPSPVDKRDRRDGYNETDTLRLLSQT
HIHSPSSLTANEMHQYGQFTKGHDPRFLQMFAPFGSQNFQIQYPSSSNVGGGGTTGNGGE
ISPSTNDQQWQTYSPQLFATAAASSGFRQQILKPQNWLQKNSVHPITRPS*

>OE6A068128P1
MWNLNYSPDQRMDEGGKGERVGSEWNSMDFSEEEDAERSGGDGNKKKSSKIFGFSVSRDC
DDDDIGPCSLGSEPPVTRQFFPVDESETTVTSGGGVAPNFPRSHWMGVKFCLSDATTVGV
LGKSAAEVSQPLKKSRRGPRSRSSQYRGVTFYRRTGRWESHIWDCGKQVYLGGFDTAHAA
ARAYDRAAIKFRGVEADINFSLDDYEEDFKQTGNLTKEEFVHVLRRRSTGFPRGSSKYRG
VTLHRCGRWEARMGQLLGKKYVYLGLFDTEVEAARAYDKAAIKCNGKEAVTNFDPSIYEE
ELGDADSSSNAFDHNLDLSLGNSASKSIGREFGDDIHHTRDQHSSSMQFEAEWRCQGLIP
EHQTQLCSTGSVKFTEMHRYGPFVKAKEPRMLPMFLPAFSSQNYQFPSSSDGGRI*

>OE6A099997P2
MWNLNYSPDQRMDEGERGKRVGSEWNFSSASLVPVEDGLSEEEEDAERSCGDGNKKRSSK
IFGFSVSHDGGDDDDDDIGPYSLGSEPPVTRQFFPVDESETTVTSGGGAAPDFPRAHWMG
VKFCQSDPNTVGVLGKSAAEVSQPLKKSRRGPRSRSSQYRGVTFYRRTGRWESHIWDCGK
QVYLGGFDTAHAAARAYDRAAIKFRGVEADINFCLDDYEEDVKQMRNLTKEEFVHVLRRQ
STGFPRGSSKYRGVTLHKCGRWEARMGQFLGKKYVYLGLFDTEVEAARAYDIAAIKSNGK
EAVTNFDPSIYEELKAPDSSSNASEHNLDLSLGNLASKPSSREFGDNVHHARDQHSSSLQ
FELEWQRRGLMPKHQVSPIDNDLQRSDVFNETDTTQLLSQTHLYSPGSMKVNEMHRYGQF
VKAKEPHMLQMFSPSFSPQNFQFSSSSNGGSIWS*

>OE6A079258P3
MSNLTKEEFVHVLRRQSTGFPKGSSKYRGVTLHKCGRWEARMGQFLGKKYVYLGLFDTEV
EAARAYDKAAIKCNGKDAVTNFDASIYDEELRGAESSNNAEYHNLDLSLGNAASKCSSRE
LEDENHDQHCSTMPFEFDLRLQGSRSELNFQNGLGQIDRRDGYNENDTLQLLSQTHLHSP
GSLKVNELHQYRQFMEGNEPRMLMFSSPNQLPSCSNGGGIRGNGEVISISTNDQLWQTKS
PQLFATAAASSGFGQQILRPQNWLHKSIVRPLTRPS*

>OE6A037406P2
MLDLNLTVDANGSIFNGTETVSEKQLELGGGQIENSGSSVSSVVNVEISNAVGDEDSCSN
PFFSSDLLKSNGEVNHYEDSENRSGNFVTKEFFPVSEGAAVAQQCQRLDLSRNYSGMVEQ
RIVAQPQQRQQVKKSRRGPRSRSSQYRGVTFYRRTGRWESHIWDCGKQVYLGGFDTAHAA
ARAYDRAAIKFRGLDADINFSVVDYEKELKQMKNLTKEEFVHILRRQSTGFSRGSSKYRG
VTLHKCGRWEARMGQLLGKKYIYLGLFDSEIEAARAYDKAAIKCNRREAVTNFDPSTYEE
EISSEAADGNHNLDLNLGIAPPCSASGQNRTIGADGFQIHCTSYSTLPENEERMHNSASA
TMETILSHGHVDVLDHPRFRKGVNSSSCPIYKGSEIGKSRELNFSANWAWQTQNPINRVP
ADPLFSAAASSGFANSSIAASSAAVLPSETISQRHYLPSVINSLYYGRS*

>OE6A055418P1
MLDLNLTIDANGSIFNGIETIWEKQHEFGGRQMENSGSSSSSVVNMEISNAVGDEDSSSN
PFFSFDILKSNGEVNDYRESENQSHGFVTTEFFPGAAVAKQCQWLDLSGNYGGAVEQRIV
AQQQPQQRQQAKKSRRGPRSRSSQYRGVTFYRRTGRWESHIWDSGKQVYLGGFDTAHDAA
RAYDRAAIKFRGIDADINFNVVDYEKDLKQIKNLTKEEFVHILRRQSTGFSRGSSKYRGV
TLHKCGRWEARMGQFLGKKYIYLGLFDSELEAARAYDKAAIKCNGREAVTNFEPSTYEEK
ISSEAAGGNHNLDLNLGIAPPCSTDGQIRTIDADGFQIHCSSDGTLPVNGARVHKYASAT
MDTQLSNGYIDVLDHPHIWKGVNSSSCPIYKGSAIEKSMDFHYSADWAWQTQNPINGVPT
EPLFSTAASSGFANSSIAASSAAVHTSETVSRHHCLPSVTNSPYYGMS*

>OE6A105872P1
MLDLNLTIDANGSIFNGIETIWEKQHEFGGRQMENSGSSSSSVVNMEISNAVGDEDSSSK
PFFSFEILKSNGEVNDYRESENQSHGFVTTEFFPGAAVAKQCQWLDLSGNYGGAVEQRIV
AQQQPLQRQQAKKSRRGPRSRSSQYRGVTFYRRTGRWESHIWDSGKQVYLGGFDTAHDAA
RAYDRAAIKFRGIDADINFNVVDYEKDLKQIKNLTKEEFVHILRRQSTGFSRGSSKYRGV
TLHKCGRWEARMGQFLGKKYIYLGLFDSELEAARAYDKAAIKCNGREAVTNFEPSTYEEK
ISSEAGNGAGGNHNLDLNLGIAPPCSTDGQIRTIDADGFQIHCSSDGTLPVNGARVHKYA
SATMDTQLSNGYIDVLDHPHIWKGVNSSSCPIYKGSAIEKSMDFHYSADWAWQTQNPTNG
VPTEPLFSTAASSGFANSSIAASSAAVHTSETVSRHHCLPSVANSPYYGMS*

>OE6A031451P2
MLDLNLTSDANESITNLGEMVLEKHLDGSGGQMENAGSYNSSVANVDTTSSAGDGDSCDN
QFIGFDIWKGNGDYEESEYQSGVSVTKEFFPVTAEGTSAARHCQWSDVSGNYGGVAAQRT
ISQQKQQIKKSRRGPRSRSSQYRGVTFYRRTGRWESHIWDSGKQVYLGGFDTAHAAAKAY
DRAAIKFRGINADINFNVSDYEEELSQIMNLPKEEFVQTLRRQSTGFSRGSSKYRGVTLH
KCGRWEARMGQLLGKKYIYLGLFDSEIEAARAYDKAAIKSNGKEAVTNFEPSTYEEGISS
EAENGGMSHNLDLNLGIAPPHLADGQNMSIDAKSFQLHSGSEGLPENRAWFSHKCSTLLQ
PQTELYYCVNM*

>OE6A031619P3
MLDLNIEAVSVDSSKCEDETDYHREMGITPIIAVAEGDSLTSTTTSATNNAVEADEQDTN
SSTRRNSLSTLNFSILKTNIIEISDDQNNDDKKSSRELQLFPTSANDAASPSAASMAKFW
LNLSVPEVPGELGNHKALRQQPPAVKKSRRGPRSRSSQYRGVTFYRRTGRWESHIWDCGK
QVYLGGFDTALAAARAYDRAAIKFRGVDADINFNISDYDVDMEQIKNLTKEEFIHTLRRQ
STGFARGSSKYRGVTLHKCGRWEARMGQYLGKKAYDKASIKSSGGDAVTNFEPGIYEKEI
NMAAGGGVSGNSLDLNLGISCSSVGVQGNENTRNVHFQYGSCELRDGKKTKGESSSNAHD
LATAPKYAPLWTGERATGMNTEANALPVFSNWPWKMQNIGVITPVPLLSSAASSGFSSVT
IPPSNSVESSNIPTTISCYTFQRPPTTLDYK*

>OE6A064439P1
MSLYVNSRQREYRKREEEMFDLNLSIDLNGKVNFEEDESPVTSTSSVVNGEGSSTCSPET
SYTFNFAILDRNSPVVKDRGLTVPEPVTRELFPMSEIGELNMGKANNNVDQRRKLVQQQK
LQVQHVQPKKSRRGPPSKSSQYRGVTFYRRTGRWESHIWDCGKQIYLGGFDTALAAARAY
DKAAIKFRGVDADINFNLGDYEEEINQMKNLSKEELVQILRRHSTGFSRGSSKYRGVTLH
KCGRWEARMGQFHGKKAYDKAAIQCNGRDAFTNFESSKYDRESMLEGSQNDLDLNLGIST
SSPKENECLALPQFQPYDVQNARKSKMDSPLKGLPLTTSRAGLNPNLVSFCEERVAEKRV
EVSQGLPYWGWRVDSQAIPMAIFSPAASSGFSSPATTSLSTDKSLYHLQTRPPP*

>OE6A012609P1
MFDLNLTLNSNDKEKKFGKGCEQEFPVTSNSLVVNSEETASNEDTCFTRESTGTGETASV
AFNFGILGGNDPYEKDHDRGLILTKTEMVTQELFPTGQLSELNLGSRSNISLDLSFNPDS
SQQARPEMVVQHQQQEVQQMKARKSRRGPKSKSSQYRGVTFYRRTGRWESHIWDCGKQVY
LGGFDTDLSAARAYDRAAIKFRGVDADINFNLADYKEDMKQMKNLSKKEFVQTLRRQSTV
FSRGSSKCRGVNLQKCGSWEARMGQFLGERAYDKAAIKSNGSDVINFDPSTYDGKMISKP
HNEGSPHDLDLNLGISTSSPLKESGSLFSYQFPPCYVQNTGQLKLRSDNSGTRVDNPLTS
EYSVLWAGVNPNFVPNYKEQEVISSQGQQEWAWQVHGRVVSTPMAMVSAAASSGFSLPAT
TSFFTAPNSSPEVSNPSAVNMCFASYSGTSTNTSRNCLQMKPSLPPP*

>OE6A073355P2
MLDLNIEAAVSVDSSTWDDETDYHRKVGITPIITVPDGDSVTSTTTSATNNNVAADEQDT
NSSTRRNSLSTLNFSILKTNIIEIADDQSNNNYKNSGNKLQLFSTSASDLASPSAASSAK
FWLNLSVPDVPGELGIYKAPHQQPPSVKKSRRGPRSRSSQYRGVTFYRRTGRWESHIWDC
GKQVYLGGFDTAHAAARAYDRAAIKFRGVDADINFNISEYEEDMEQMKNMKKEEFVQILR
HPSTGFARGSSKYRGVSLHKSGQFEARMGQYLGNKAYDKAAIKSRGLEAVTNFKPGIYEK
EINIAAGAGGIGTGNSLDLNLGISCPSDGLKGNGNTRNMHFHYGSHKLPDGKRIKVESSS
AAHGLVTAPKYAPMWTGVYSGFTLNNKERATGMNTEANALPVYSNWPWKMQSHGVITPVP
LLASAASSGFESIHVPTTISCDTLQHPPTTLD*

>OE6A121142P2
MFDLNLSIGSNWKENFEEDESPVTTASWVVSGEEGSSYNGTWSTRASPEKSHTFNFGILN
RDDPVEKDCNLTESEPVTRELFPVSGLCELNMGRPNNNGNGNWINQQVVPSKLVHQQVQH
VKPKKSRRGPPSRSSQYRGVTFYRRTGRWESHIWDCGKQIYLGGFDTALAAARVYDRAAI
KFRGVDADINFNLSDYEGEINQTRSLSKEEFVHRLRGRSTGFSKYKCGRWEARITGKKAA
IQCNGRDAESSKYEEESISEPRNEGSQHHLDLNLGISTTSPNENESLALLQFQPGVVQNA
RKSKMDPPLKGTSLAGLNPKSVSSYEVNLSTVLRFIGAISVLYAPFLPHLLAIKTSNLEN
NSLI*

References

Sievers F., Wilm A., Dineen D., Gibson T.J., Karplus K., Li W., Lopez R., McWilliam H., Remmert M., Söding J., Thompson J.D. & Higgins D.G. (2011) Fast, scalable generation of high-quality protein multiple sequence alignments using Clustal Omega. *Molecular Systems Biology*, **7**, 539.
